# Current Practices and Opportunities for More Sustainable Public Food Procurement: A Qualitative Study among Danish Municipalities and Regions

**DOI:** 10.3390/foods12101975

**Published:** 2023-05-12

**Authors:** Anne Dahl Lassen, Anne Vibeke Thorsen, Ellen Trolle

**Affiliations:** National Food Institute, Technical University of Denmark, DK-2800 Kgs. Lyngby, Denmark; avth@food.dtu.dk (A.V.T.); eltr@food.dtu.dk (E.T.)

**Keywords:** public food procurement, sustainable food, foodservice, sustainable and healthy food, sustainable cities, organic food, green public procurement

## Abstract

Public food procurement has been emphasized as a powerful tool to promote a healthier and more sustainable food system, but there is still a long way to go to reach full potential. This study aimed to investigate practices and opportunities for sustainable and healthy public food procurement. A qualitative cross-sectional study was performed among Danish municipalities and regions stratified and randomly selected to cover standard practice (*n* = 17). In addition, interviews were performed among selected best-practice municipalities (*n* = 5) providing examples of ambitious goals and well-defined processes for obtaining sustainable food procurement. Large differences were observed in the cross-sectional analysis in relation to policy support and goals for sustainable food procurement, including organic purchase. Generally, there was a great attentiveness to reduce food waste and many valued the use of local food, especially among rural municipalities, whereas experience with climate impact reduction and shifts towards more plant-based menus was still in an early implementation stage. Results suggest a possible synergy effect between the use of organic food and efforts to reduce food waste and climate impact and emphasize the importance of local government policies to promote healthy and sustainable food procurement. Enabling factors to move sustainable food procurement forward are discussed.

## 1. Introduction

Sustainable public food procurement has been emphasized as an important strategic tool to address our current sustainability challenges and bring the world closer to reaching the UN 17 Sustainable Development Goals (SDGs) by 2030 [1]. A range of SDGs are interconnected with the performance of food systems, including SDG #2 (Zero hunger), SDG #3 (Good health and well-being), SDG #6 (Clean water and sanitation), SDG #13 (Climate action), SDG #14 (Life below water), and SDG #15 (Life on land) [2]. SDG #12 (Responsible consumption and production) addresses public procurement directly (12.7 Promote public procurement practices that are sustainable, in accordance with national policies and priorities) [3].

Sustainable public food procurement helps to orientate businesses—both the agriculture and food industries—towards more sustainable practices and product development [4]. In addition, meals provided by public institutions may inspire children and adults towards eating healthier and more environmental sustainable diets by experiencing tasty meals and may further help normalize the sustainability agenda in the population [5,6,7].

Public food procurement and services relate to both the purchasing of foods and foodservice operations, whether self-operated or contract managed. It applies to different public settings, for example schools, child-care centers, hospitals, nursing homes, public-sector worksites and other venues where meals are provided. In 2021, the World Health Organization (WHO) presented an action framework aimed at supporting the development and implementation of food procurement and related service policies that encourage a healthy diet while incorporating sustainable practices [8]. Many municipalities and local authorities worldwide have set targets to ensure healthy and sustainable procurement and meals [9,10]. The Brazilian School Food Program is an example of a public procurement policy that has several benefits. It is mandated, among other components, to purchase 30% of its supply for meals from family farmers [11,12].

In Europe, to promote healthy and sustainable diets in institutional catering, the “Farm to Fork” strategy states that the best way to set minimum mandatory criteria for sustainable food procurement will be determined [13]. The European Public Health Alliance suggests that procurement strategies throughout the European Union should contain, at least, criteria on nutritional quality of foods and menus, the share of organic products in procurement (e.g., 20–50%), the share of foods from other quality and/or sustainability schemes, and the share of plant-based menus offered [14]. Organic farming is promoted explicitly in the Farm to Fork strategy and in March 2021, the European Commission announced an action plan for the development of organic agricultural production, including expanding procurement by public institutions [15]. Likewise, the Danish government has suggested developing minimum mandatory criteria for sustainable food procurement in the government’s strategy “Green Procurement for a Green Future” [16].

Sustainable food procurement is a complex concept, which relates to a variety of practices. The European Union Green Public Procurement program provides a voluntary framework to encourage public bodies to procure sustainable goods and services, including the use of organic food products, sustainable marine and aquaculture food products, animal welfare considerations, fair and ethical trade products and more environmentally responsible vegetable fats and agricultural products labelled with geographical indications. Criteria proposals for catering services include, e.g., more plant-based meals, minimizing food and beverage waste and improving the use of resources such as energy and water [17].

Although sustainable public procurement is gaining momentum globally and locally, there is still a long way to go. The IPES-Food panel (The International Panel of Experts on Sustainable Food Systems) states that the procurement policies have been insufficiently used to drive production shifts [18]. Moreover, Swensson et al. states that despite the increasing recognition and potential of public food procurement, it remains an underexplored topic [19], and there is a is a lack of research that covers a wider range of sustainability aspects. This is particularly the case for settings other than the school environment [20].

Thus, the objective of this study was to provide insights into food procurement in Danish municipalities and regions, i.e., the use of organic food, local and seasonal food, climate-friendly and healthy menus and food waste minimization initiatives. Current practices as well as best-practice cases were investigated in order to best identify how further development can be achieved and promoted and to serve as inspiration for authorities and institutions worldwide that provide meals to the public such as hospitals, schools, child-care and nursing homes.

## 2. Materials and Methods

**Figure 1:**
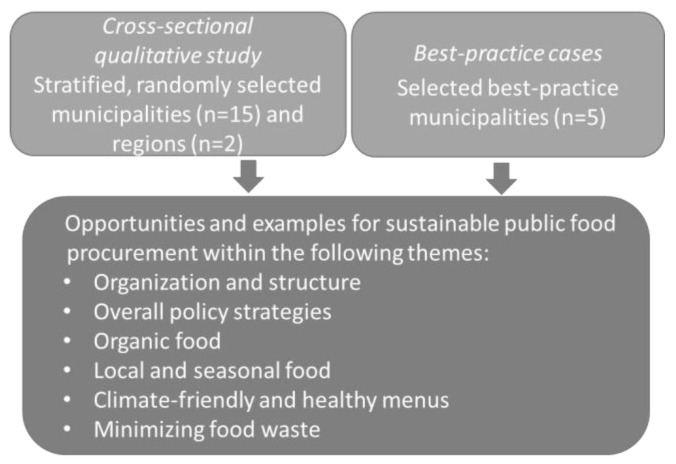
The qualitatively design and themes of the total study.

In Denmark, public hospitals are operated by a total of five regions, and a total of 98 municipalities are responsible for a number of health and social services including schools, child-care and nursing homes. Figure 1 illustrates the design of the total study, consisting of two studies based on semi-structured qualitative interviews. The cross-sectional study was conducted among regions (*n* = 2) responsible for hospital operation, which were selected among the total of five regions by use of a random number generator, and among municipalities (*n* = 15), where selection was done by first dividing all the 98 municipalities into the 5 Region Areas in Denmark and further dividing each group into three different groups to cover urban municipalities with unknown objectives for organic food procurement, rural municipalities with unknown objectives for organic food procurement and municipalities known to have objectives for organic food procurement (see Table 1). This was done to reflect the diversity in food procurements of municipalities, i.e., to ensure representation from all regions of the country from both rural and urban municipalities because of different access to local farmers and to include municipalities both with and without organic objectives, serving as an indicator for a general focus on sustainability and political support. One municipality was randomly selected from each of the groups and regions by use of a random number generator to be included in the study. A total of three municipalities declined to participate due to busyness on account of COVID-19 and were replaced by other municipalities having the same profile with regard to region and organic procurement objectives.

The second study was conducted as a best-practice case study with selected municipalities, having sustainability aspects included in their food procurement agreements (*n* = 5). The municipalities were chosen in order to cover different parts of Denmark: the four largest Danish municipalities (Copenhagen, Aarhus, Odense and Aalborg) and one medium size municipality (Skanderborg).

Opportunities and examples of the integration and documentation of different aspects of health and environmental sustainability within public procurement and food services were gathered from both studies.

The studies were conducted from November 2021 to February 2022 as online semi-structured interviews. The semi-structured interview was chosen for its flexibility, which allows for the use of open-ended questions and the exploration of emerging sub-themes [23]. The study was conducted in accordance with the Declaration of Helsinki. It did not require ethical approval. Participants were informed about the purpose of the research via e-mail at the beginning of the interview and were at the same time informed that the interviews conducted in the research would be recorded. The participants of both studies were asked for their consent to participate in the study under these conditions and accepted this verbally. Detailed notes were prepared on the recordings afterwards, and all the notes were reviewed and accepted by the interviewees. In the cross-sectional study, all participants were anonymized. If possible, interviews were done with both an officer or manager of the procurement department and a health and care officer, with knowledge of the procurement practice and strategies as well one or more food service managers to gain insight into catering practices. Interviews lasted between 30 and 60 min.

The interviews covered the following areas: organization and structure, overall policy strategies and specific initiatives with regard to requirements for sustainable food procurement, and other sustainable catering practice and documentation within the following predetermined themes: organic food, local and seasonal foods, climate-friendly and healthy menus, and food waste. Current practices were analysed using a thematic content analysis approach [24] and summarized in a descriptive text for the different themes.

In the best-practice cases, municipalities were not anonymized, and the participants were given the opportunity to review their responses and were allowed to correct or clarify their description, leading to minor changes. The interviews were performed with municipal stakeholders dealing with procurement and food services, and relevant documents were gathered.

## 3. Results

Results for the cross-sectional study and the best-practice cases are presented in Section 3.1 and Section 3.2, respectively, while Table S1 in the Supplemental Material presents examples from both studies of opportunities for setting requirements linked to sustainable procurement and other catering practices as well as possibilities for documentation.

### 3.1. Cross-Sectional Study

#### 3.1.1. Participating Municipalities and Regions

Table 1 gives an overview of participating municipalities (*n* = 15) and regions (*n* = 2) in the cross-sectional analysis. A total of 34 respondents from the municipalities in addition to 5 respondents from the regions took part in the interviews. In 14 of the 15 municipalities, the procurement division attended the interviews. In addition, the catering division attended interviews in 13 of the 15 municipalities.

Municipalities were distributed among all five Danish regions (three from each region), representing both rural and urban areas and different foci considering sustainable procurement (67% with no registered objectives for organic food procurement).

Based on the responses, close to one-third of the municipalities and one of two regions were members of the procurement network organization Partnership for Public Green Procurement (POGI). In addition, most municipalities responded that they were involved in another national and/or local procurement community or network.

All municipalities stated that the meals offered included full day menus for residents at nursing homes as well as typically warm meals to home residents through food delivery. To a variable degree, meals offered also included child-care centers, schools and town halls. Some municipalities are responsible for catering themselves while others use external suppliers, or a combination. The regions stated that the meals offered included meals delivered to patients and employees and at publicly accessible cafes at hospitals.

Within the major themes, several sub-themes were identified, as shown in Table 2.

#### 3.1.2. Overall Strategies

The participating municipalities and regions all had some reflections regarding sustainable procurement.

*“Sustainability is coming in the future, it’s common sense. In 2 years, the municipality will prioritize sustainability with new policies and strategies”*.

Procurement Officer, M6

Several of the municipalities expressed that they had initiatives underway in these areas, and several mentioned the 17 UN SDGs as the overall framework for the procurement practice and its sustainability focus. However, large differences among municipalities and regions were seen in relation to overall policy support and goals for sustainable public food procurement and meals.

In some cases, the absence of clear goals in this area was perceived by the interviewees as an obstacle to enhance sustainable procurement implementation and sustainable food production.

*“We have no requirements for the environmental sustainability and no policy to adhere to, so I cannot make demands that cost money and that the council has not approved”*.

Procurement Officer, M8

#### 3.1.3. Organic Food

Among municipalities and regions, the policy priorities in relation to organic food differed markedly. While around one out of three municipalities and regions stated that they have policy support for the purchase of organic food and already have achieved a high organic procurement share, most municipalities in this study experienced a lack of policy support for organic procurement. Nevertheless, several municipalities declared that some of the kitchens, on their own initiative, have an organic procurement share of, e.g., 10–25% or more.

*“Some have around 90% organic products, while some kitchens don’t focus on organic at all, so it ranges from one extreme to the other”*.

Health and care officer, M7

As a driver for organic food procurement, both municipalities and the regions mentioned biodiversity and product diversity. On the other hand, the economy was mentioned as a barrier for increasing the share of organic food procurement. In general, child-care centers have a higher organic procurement share compared with the elder care sector. Demands from the parent boards in child-care centers are mentioned as a reason for the higher share of organic food in some child-care centers. The lower share of organic food in the elderly care sector is partly explained by greater financial demands due to an increased proportion of meat in the meals, as the protein requirement is higher for older adults.

#### 3.1.4. Local and Seasonal Food

With regard to seasonal considerations and the use of local goods, this is valued by many municipalities and regions, e.g., locally grown strawberries and potatoes in season. In this study, this was particularly evident in rural municipalities, and to a lesser extent in urban municipalities and regions. Approximately one in four respondents expressed uncertainty regarding the opportunities provided by public procurement agreements to buy local foods. Other barriers to buying local foods were mentioned, such as the need for kitchen facilities to handle fresh, whole foods. Additionally, some respondents expressed concern that local suppliers may not be able to provide sufficient quantities to meet the needs of professional kitchens.

*“In the future, there will be the opportunity to use local suppliers, and there will be a greater focus on this going forward”*.

Health and care officer, M3

#### 3.1.5. Climate-Friendly and Healthy Menus

Rural municipalities that have not recognized an organic objective expressed that only a few initiatives had been undertaken towards more climate-friendly food procurement. Among urban municipalities, the majority had some reflection on and initiatives involving the consumption of legumes, plant proteins, or meat-free days to the extent that is was perceived as following nutritional recommendations and acceptable to e.g., older adults. More specific goals for, and initiatives to, reduce the impact on climate were mainly initiated by municipalities and regions having an organic objective. Around half of these municipalities and regions indicated that the meal composition had been changed to some extent towards a more climate-friendly composition, consisting of, e.g., less use of beef and veal and more legumes.

*“We have new menu plans with more vegetarian dishes several times a week and more fish and poultry. The elderly people like vegetarian dishes”*.

Food service manager, M12

*“We are in the process of looking at reducing the amount of beef and veal. Typically, once a week, there is an option to choose a dish based on green vegetables”*.

Food service manager, M13

The same applied to the regions where one of the regions had set specific climate-reduction focus points for meals in the canteens, cafes and hospital meals—the latter, however, with lower goals for reduction. Three of the municipalities and regions expressed a need for the suppliers to provide climate impact data, preferably at the product level as well as at the product group level, in order to be able to document progress with regard to climate. As a barrier, some of the municipalities and regions expressed concern about the nutritional composition of the meals especially for older adults and sick people if the menu was changed to a more climate-friendly one.

The majority of the municipalities stated that they followed the Danish official dietary guidelines; however, it is unclear whether they document this on a regular basis.

#### 3.1.6. Food Waste

In general, there is a great interest and focus on reducing food waste. This has been a particular focus area for kitchens that has changed towards purchasing more organic food, as the organic price premium in many cases has been partly covered by reducing food waste. However, municipalities without organic procurement objectives also stated that food waste has been a focus point for some years not least for economic reasons.

*“We have adopted a new dietary concept in recent years, starting around 2018, while simultaneously reducing our food waste to less than half”*.

Procurement officer, R1

In addition, the focus is on setting requirements to reduce food waste at the supplier’s level. One region (R1) has the ambition of reducing food waste by 20% over a 4-year period. As an example of an initiative, a municipality mentioned that a food waste analysis implied that nursing homes now order smaller portions. Another municipality mentioned that food waste in child care has been reduced through planning, avoiding making too large portions, and reusing leftovers in other dishes or in baked goods.

### 3.2. Best-Practice Cases

#### 3.2.1. Copenhagen Municipality

In 2019, in the Municipality of Copenhagen, 84% of the food served from public meals was estimated to be organic. To achieve this, the municipality has focused on training and upskilling food professionals with the support of consultants. The municipality’s Food and Meal Strategy raises the level of ambition so that each individual kitchen by 2025 must achieve 90% organic in the meal served, with a few exceptions [25]. In addition, the strategy sets a target to reduce the carbon footprint from public meals by 25% by 2025 relative to 2018, while ensuring nutritional requirements, culinary quality and keeping organic food purchases at a high level. The municipality has provided guidelines for overall food composition and practical application in menu planning, i.e., rules of thumb for the number of servings over a one-week period of time for each main food group for different groups of users [26,27]. Moreover, the municipality has published a climate-friendly database of recipes and offered training and support to the staff in the municipalities kitchens to help ensure compliance with the municipality’s guidelines [28].

The interviewees further expressed that the municipality has included further social and environmental criteria into their food procurement using the 17 UN SDGs as a guide. Regarding food procurement, the Municipality of Copenhagen has created a climate weighting procedure that reflects which products are important within the implementation of the municipality’s benchmarks and guidelines. Consequently, the municipality has achieved more competitive prices on the foods that the kitchens need to provide more of, such as nuts. Another requirement incorporated in the food procurement call for tenders is that it must be feasible for the individual food service units to monitor the carbon footprint of the unit’s purchases divided into different food categories.

In the Municipality of Copenhagen, limiting food waste is a high priority in creating environmentally and climate-responsible meals, including preventing food waste from the suppliers in collaboration with the municipality. In 2021, an analysis was completed of the extent and type of food waste in the public kitchens, which will form the basis for the setting of future reduction targets and action plans.

Other focus areas that were expressed included seasonality, securing food product diversity, requirements to support the demand for responsibly produced soy, the phasing out of non-certified palm oil and prioritization of Fairtrade labeled products and sustainable fish, including sustainably certified fish and excluding the fish species that do not appear on the list of World Wide Fund for Nature’s endangered and soon-endangered species. Finally, requirements are set for aspects such as packaging, transport, delivery, etc.

#### 3.2.2. Aarhus Municipality

The Aarhus Municipality is using the 17 UN SDGs as a platform in relation to the three dimensions of the “bottom line”—environmental value, economic dignity, and social value.

The Aarhus Municipality has set three goals by 2025—a 25% reduction in the carbon footprint of purchased food, reduction of food waste and more climate-friendly foods in the food procurement. It was expressed that this should be done without compromising on organic food purchases, taste and nutritional quality, just as it was stated that seasonal matter should be used as well as enhancing the diversity of food products and consumption. It was expected that the suppliers soon will be able to provide climate reports to the individual food service units.

Child-care centers, schools, leisure, and youth school facilities that offer children and young people food and drink must set a goal of 60% organic food procurement. In child-care centers having in-house food production, the goal is 90% organic food procurement. The Aarhus Municipality started to follow the Danish official recommendations where possible. Thus, the focus is serving more vegetables—many greens, more beans, lentils and legumes, more nuts and seeds, more fruit, whole grains and potatoes and less red and processed meat.

The Aarhus Municipality works closely together with their food supplier on climate-friendly foods, such as locally grown lentils and quinoa. In addition, projects are initiated with entrepreneurial companies to focus on local sustainable initiatives, and the municipality is involved in testing and developing local products, for example micro-greens, e.g., cress, gourmet mushrooms and fermented products, in collaboration with start-up companies. In general, it is considered that collaborations and partnerships are central to achieving the municipality’s goals.

An important goal is to develop the competences of food professionals through, among other things, digital courses and in-person education courses. Moreover, emphasis is placed on making it easier to find and be inspired to purchase climate-friendly foods through advertisements on climate-friendly items and making information available on, e.g., carbon footprint at the point of purchase. Finally, requirements are set for aspects such as packaging, transport, delivery, etc.

#### 3.2.3. Odense Municipality

The Odense Municipality has developed a method and process that means that all relevant public food procurement and suppliers are checked and compared with the 17 UN SDGs by the municipality. Therefore, as expressed by the interviewees, the Odense Municipality’s process supports an inclusive market dialogue, where suppliers are helped to identify SDGs to which they contribute and to overcome the barriers that sustainability criteria add to the contracts. Sustainability is assumed to include culture, economy, climate, nutrition, and packaging. In addition, the Odense Municipality has decided, based on the SDG# 14, to help stop overfishing, restore fish stocks and to protect marine ecosystems. Consequently, the central kitchen of the Municipality of Odense does not serve, for example, salmon and tuna, as the fishing of these is not sustainable.

In addition to the overall approach to the 17 UN SDGs, the municipality has specific policy goals that 60% of the food the municipality procures should be organic and 30% of it should be grown locally [29]. The Odense Municipality has for some years been working with using local food products to focus on promoting local business in addition to sustainability. The Odense Municipality has several smaller trade agreements for the procurement of local organic goods, e.g., vegetables and fruit, flour and grains, making it possible to work with local food suppliers in addition to the overall purchase agreement. However, it has required advice and practical support to make local suppliers capable of meeting requirements for deliveries, e.g., to deliver electronic invoices. The principles for food purchasing and menu composition include the following: choose organic food and food from local vendors; use whole raw materials; use seasonal vegetables and fruits; eat less meat; and eat more fish, beans, lentils, peas, potatoes and grains.

#### 3.2.4. Aalborg Municipality

In the Aalborg Municipality, public food procurement considerations include sustainability, organic food, seasonal commodities and local commodities, using the 17 UN SDGs as the overall framework for this work. The municipality has a particular focus on three of the global goals: Goal #7 on sustainable energy, Goal #8 on decent jobs and economic growth (social responsibility) and Goal #12 on responsible consumption and production (environment and climate).

The share of organic products of total purchase has increased from zero percent in 2013 to current 67%. Price increases during this process have been avoided by reducing food waste (with additional costs in terms of training staff). Reducing food waste is an ongoing process where, among other things, the focus has been on avoiding over-requisitioning food at nursing homes. The municipality’s vision is to continue the organic “journey”, where beans, lentils and chickpeas will be included to a greater extent.

The Aalborg Municipality practices knowledge-sharing with other municipalities. In addition, market dialogues are conducted with suppliers. In the budget for 2022, funds have been set aside to work at measuring sustainability and to support the various stakeholders in relation to green procurement.

#### 3.2.5. Skanderborg Municipality

In 2020, the Skanderborg City Council adopted a climate, energy and resource policy with objectives and efforts, among other things, to reduce the food carbon footprint and reduce food waste [30]. With a grant, the green ambitions have resulted in several courses for the staff in the municipality’s public kitchens. The internal goal of the courses includes the following: the organic percentage must be increased by at least 10%, food waste should be reduced by at least 25%, the proportion of purchased beef should be reduced by at least 50% and the proportion of purchased lentils, beans and legumes should be increased by at least 50%. It was specified that the goal of organic procurement was developed by the employees in the kitchens. The vast majority of the kitchens have the Danish Organic Cuisine Label [31] of bronze, which means purchasing 30–60% organic food, some have the silver label, which means purchasing 60–90% organic food and a few have the gold organic label (90%), which means purchasing minimum 90% organic food.

## 4. Discussion

The present study shows that across municipalities and regions in Denmark, food procurement covers multiple interconnected sustainability topics linked to the UN’s 17 Sustainable Development Goals. The focus of this study was to provide insights into both usual practices and best-practice regarding food procurement and other catering practice covering the following main areas: organic food, local and seasonal food, climate-friendly and healthy menus (e.g., more plant-based menus) and minimizing food waste.

Best-practice municipalities in the present study provided illustrations of ambitious frameworks and goals for sustainable food procurement. Some of the goals have already been attained while others are still being implemented. Likewise, the cross-sectional analysis showed that most municipalities and regions have taken steps and started several initiatives to move towards more sustainable food procurement practices. However, results suggested that for some municipalities the development was hampered by a lack of clear policies and guidelines and consequently had less well-defined processes for sustainable public procurement.

### 4.1. Organic Foods

The global market for organic food and drink sales continued to grow globally in 2021; however, the growth is at a slower rate compared with that observed in 2020 [32]. In 2022, rising food prices have made consumers more price sensitive and demand for premium products, including organic foods, has been negatively affected in Europe [32]. As a driver for purchasing organic foods, municipalities mentioned issues such as enhancing the diversity of food production and biodiversity. Adding more organic food to the menus may benefit local biodiversity, soil quality and ecotoxicity levels among other things due to, e.g., reduced pesticide use [33], and may also contribute to improved animal welfare [34].

Sustainable procurement requirements mentioned in the present studies included ensuring availability of an organic product supply by establishing requirements for a fully organic variety. Some of the participating municipalities had set requirements for a minimum organic share of purchased food. The municipality with the most ambitious goals is the Municipality of Copenhagen with the policy ambition by 2025 to reach 90–100% organic throughout the procurement of the 900 kitchens that produce public meals across the city, with a few exceptions. The total organic percentage for 2020 was 84% [35].

Schleifer et al. points out that despite the growing number of cities that have set targets for organic food in public food procurement, studies that quantify the actual share of organic products in such systems are still rare [36]. An example of a city that has quantified its organic share is Vienna, Austria. Vienna estimates that one-third of the city’s food preparations for older adults and hospitals use organically farmed food; in schools and kindergartens, this has reached 50% [37]. In Denmark, the suppliers may assist public kitchens in calculating the organic share of the purchased foods, which eases the burden of documentation for the public kitchens [38].

In Denmark, the use of organic food procurement in public kitchens has a long history of implementation [39]. The additional costs in public kitchens caused by the conversion toward more organic products have been largely covered by buying seasonal food products, reducing food waste and purchasing less processed food products [39,40]. In line with this, initiatives in the area of organic procurement together with the prevention of food waste were among the most frequently sustainability goals mentioned by the municipalities and regions. However, the policy priorities in relation to organic food differed considerately. It has been shown that policy goals promoting organic food have a positive effect on a municipality’s share of organic food purchases and this effect increased with the ambition level [41]. In some municipalities in the present study, the conversion of more organic foods was to some extent driven by the catering units on their own initiative or based on demands from the parent boards at child-care centers.

The establishment of the Danish Organic Cuisine Label, in a study by Sørensen et al., was found to contribute to a maintained or increased organic food percentage within the public kitchens [39]. This may be attributed to the strong commitment expressed by kitchen managers who have obtained the Danish Organic Cuisine Label to maintain their organic procurement level despite possible budget constraints, as well as the increased awareness of the kitchens use of organic products among both customers and decision-makers.

### 4.2. Local and Seasonal Food

In a global view, the promotion of local food procurement and improvement in local farming and production may achieve progress towards the UN’s SDGs, particularly SDG #2 (Zero hunger) [42]. A study comparing the sustainability of local and global food products concluded that local products perform better in areas of sustainability that are mostly concerned with quality and place (e.g., territoriality, nutrition, animal welfare and biodiversity) whereas global products outperform local products in areas related to quantity management (e.g., affordability and food safety) [43]. In line with this, Tubiello et al. have highlighted the increasingly important role that food-related emissions related to pre- and post-production processes play along food supply chains (including transport among other things such as food waste disposal) [44].

The use of locally and seasonally grown goods was in the present study found to be valued by many municipalities, notably to diminish “food miles” and support local economies, including small and local-regional farmers, traditions and culture in food production, as well as ensuring diversity in food products. Likewise, a recently published review on local food procurement policies and practices in primary and secondary school food service found that perhaps the most common initiator or motivation for participating in local food procurement is a sense of social responsibility [45].

The examples of requirements for sustainable procurement and catering practices in the present study were having demand for specific varieties of e.g., vegetables, requirements for the use of specific product groups locally, e.g., local seasonal vegetables and setting requirements and targets for a minimum share of purchased food that must be from local sources, e.g., approx. 30% of total food procurement by the Odense Municipality. Likewise in Brazil, the National School Feeding Programme requires 30% of the budget to be used to purchase food from family farms [12].

Nevertheless, several of the interviewees in the present study expressed uncertainty about the local suppliers’ ability to accommodate deliveries, as well as a need for facilities to handle the products that are less processed. Galt and Elbrecht recommend simplifying procurement procedures, actively engaging small and medium enterprises, and breaking large tenders up into smaller bids, all of which could help to remove the barriers these enterprises currently face to participating in these procurements [5]. The European Public Health Alliance suggests that the European Commission shall clarify conditions under which local procurement can be enabled while ensuring the functioning of the internal market [14].

### 4.3. Climate Consideration, Nutrition and Meal Composition

An important starting point for the transformation towards more climate-friendly food procurement is the replacement of animal-based foods, especially beef and lamb meat, with plant-based products [46,47]. The studies revealed that many municipalities and regions already have set requirements for reducing the climate impact of their food purchases or are working on considerations in this area. On the other hand, experience with climate impact reduction with regards to food procurement is still in a fairly early implementation stage.

From the studies, it appears that the municipalities having worked with organic conversion have good qualifications for initiating projects related to reducing carbon footprint. The kitchens have become accustomed to make changes and new routines to improve sustainability of the meals they serve. Furthermore, more than half of these municipalities and regions indicated that they have already reduced the amount of meat in the menus to some extent. The possibilities for making more climate-friendly menus include reducing serving sizes of meat, offering dishes with a high environmental impact less frequently, creating new recipes or simply by replacing a specific ingredient or part of an ingredient within an established recipe with a more climate-friendly option [27,48].

The majority of the municipalities stated that they follow the Danish official dietary guidelines, i.e., the Danish Climate-Friendly Dietary Guidelines [49,50] and Recommendations of the Danish Institution Diet [51]. However, it is unclear whether they document this on a regular basis. The Danish Climate-Friendly Dietary Guidelines for Meals in kindergartens, schools and workplaces were launched after the study interviews were conducted [52].

The interviews revealed a concern about the nutritional composition and acceptability of the meals to especially vulnerable older adults in nursing homes if changing the menu to a more climate-friendly menu. Moreover, there is a need to take meal preferences and recognizability into account for this consumer group [27]. When changing the meals to make them more healthy and environmental sustainable, a holistic view on the whole menu is recommended. When replacing specific ingredients, the focus must be on replacing with less climate-intensive ingredients that fulfill similar nutritional and culinary function in the dish, e.g., butter replaced by rapeseed oil and meat by legumes. The replacement of a part of the meat in a meat dish by plant-based ingredients has been proven to be a consumer-accepted strategy [53]. Likewise, a partial replacement of wheat flour in bread with pulses to increase protein content in a cereal product has proven to be successful [54]. In addition, to ensure the nutritional adequacy of the total menu more adjustments may be needed. This may include increasing the content of whole grain products and dark green vegetables. Moreover, products such as legumes, nuts and seeds, including tahini and peanut butter, can be integrated into other parts of the meals, e.g., in dressings, sauces, soups, cakes and in-between meals and drinks [27,46].

Examples of requirements in the calls for tenders for sustainable food procurement in the municipalities included information on climate data at food level and aggregated for each catering unit from suppliers, as well as competitive prices for the more climate-friendly foods. Examples of requirements for sustainable catering practices included the presence of more plant-based meal options and nudging towards these meals. Furthermore, in 2022, the Aarhus municipality decided to impose an internal climate tax on selected food groups, such as beef, lamb, ready meals with meat, and juice and soft drinks purchased by the municipality in connection with a new food procurement agreement being implemented later in 2022 [55].

Worldwide cities are committed to reducing climate impact of their food procurement. From 2021 and onwards in Ghent, Belgium, the meat consumption in the city’s school canteens is reduced by 50%. Protein sources consist of 50% animal-based and 50% plant-based protein [10]. Similarly, in Oslo, Norway, the goal is to cut the meat consumption in half in the municipality’s canteens and institutions by the end of 2023, and in Helsinki, Finland, the goal is to increase the percentage of vegetarian food served in public buildings, and halving the use of meat and dairy in the food procured by 2025 [10].

### 4.4. Minimizing Food Waste

The UN’s SDG #12.3 seeks to halve the global food waste at retail and consumer levels. In general, the municipalities and the regions in the study had a great interest and focus on reducing food waste, especially among those having converted towards using more organic products. This is in line with a former quantitative study among canteens at schools and workplaces indicating that canteens with higher organic procurement are first movers on more comprehensive waste reduction. The study showed that canteens using organic procurement had more priorities in optimizing, especially in the use of raw foods (i.e., using the entire product), but also adapting portion size/serving bowl size and reusing excess production and leftovers compared with those not using organic food procurement to the same extent [56].

Several municipalities report requirements for reducing food waste during catering practice, e.g., 33% in the Aarhus Municipality. Examples of sustainable procurement criteria included requirements for the supplier’s handling of waste, e.g., the supplier must have policies for minimizing food waste, possibility of highlighting foods when purchasing in order to avoid food waste and requirements regarding e.g., reducing food waste through planning and portion size control. Other food waste reduction initiatives included dialog with nursing staff to avoid over-requisitioning of food and information directed at consumers on what and how much plate waste is discarded.

Sundin et al. detected a “vegetarian paradox” based on quantitative food waste data combined with interviews with kitchen staff at 10 Swedish primary and secondary schools. Vegetarian options were considered to be unpopular, but several vegetarian options were among the most popular choices and generated less waste, including vegetarian nuggets, red lasagna, potato pancakes, tacos, and soups [57]. Consequently, school-catering units should focus on serving popular nutritious meals, including popular plant-based options, as part of efforts to make school meal schemes more acceptable and sustainable. Malefors estimated that food waste in the Swedish public catering sector was reduced by 25% between 2016 and 2020 [58].

### 4.5. Enabling Factors and Opportunities for Implementation

To move sustainable food procurement forward, different enabling factors were highlighted by the participants in the present study. Studies on establishing an easy-to-use monitoring system are needed to document progress, including documentation on e.g., climate impact and food composition at the unit level, e.g., provided by wholesalers. The increasing accessibility of digital food data offers many opportunities. There are, however, challenges to be solved, with respect to the quality, uniformity and accessibility of these data.

Procurement officers may be supported by means of working together to build on the lessons learned from existing initiatives [5]. The WHO European Office for the Prevention and Control of Noncommunicable Diseases has developed a manual for public procurement officers across the WHO European Region. This manual guides procurement officers in adopting practices that promote a healthy and sustainable diet [59]. This includes market dialogue that is important to encourage food producers to develop healthy and sustainable products and motivate wholesalers to promote certain products. The experience of the Copenhagen and Odense municipalities shows that market dialogue is important to help suppliers overcome the barriers that sustainability criteria add to contracts and to build strong partnerships [60]. Requirements can be set according to national priorities and can evolve over time. It is important to have a careful balance between ambition and pragmatism, or else there will be no bidders on the tender. The market managers must be able to discuss and reflect on the tender, and possibly be open for innovative, stepwise approaches to encourage healthier or more sustainable procurement [61].

In addition, efforts should focus on ways to increase staff skills, motivation and ownership. Key elements include empowering and educating the staff and getting everyone involved proactively in the processes [62,63]. In line with this, food procurement policy should be accompanied with wrap-around initiatives to gain support for changes and creating working relationships with e.g., customers and key stakeholders [64]. Messages should be stated in positive culinary terms, as the major motivator to make dietary changes is unlikely to be the health benefits, but rather sensory appeal and attractiveness [63].

Finally, on the government level, the EU Food Policy Coalition’s Manifesto on sustainable public procurement calls for establishing minimum standards for public canteens across the EU [65]. The European Union and Member States shall ensure adequate support, including financial, by drawing on existing European Union financial mechanisms, to enable the achievement of minimum requirements [14]. This aligns with the principle of establishing “pull” provisions setting incentives for food systems actors to go beyond minimum requirements.

### 4.6. Strengths and Limitations

A limitation of the study is that it was conducted in Denmark, which means that the results may not be directly applicable to all international contexts. Nevertheless, it is possible that the study can serve as a source of inspiration for authorities and institutions worldwide, both within and outside Europe, regarding a wide range of aspects related to the sustainability of public food procurement, i.e., organic food, local and seasonal food, climate-friendly and healthy menus and minimizing food waste. Furthermore, it is a strength that both front-runner municipalities and stratified, randomly selected municipalities were included to elaborate the understanding of how municipalities can expand sustainable food procurement and catering practice and to better understand the changes needed and challenges across different settings with different procurement practices. This study is based mainly on interviews and therefore may not necessarily reflect actual practice in all aspects. However, whenever possible, additional data sources such as procurement and food service policies were gathered to verify and expand the knowledge obtained from the interviews.

The European Union Green Public Procurement program includes aspects such as the use of resources (energy and water) and impact on biodiversity losses [17] not encompassed in the present study. Likewise, other sustainability aspects related to packaging, transport, social responsibility, and labelling at product level are not covered here, although these aspects are also given great importance by many of the respondents. Among other things, brands and standards may pave the way, e.g., fairtrade products and certified fish. Moreover, several municipalities mentioned the goal of purchasing fewer processed products, especially highly processed products.

## 5. Conclusions

Current practices as well as best-practice cases provided insight into further development of sustainable food procurement that are reachable and can be promoted at public settings, such as hospitals, schools, child-care and nursing homes. This may provide inspiration for setting further requirements within sustainable procurement and setting minimum criteria for sustainable public food procurement.

Best-practice cases revealed ambitious goals such as up to 90% organic food procurement, 30% food from local sources and a reduction of food waste as well as reducing the climate impact by 25%. Large differences among municipalities and regions were seen in the cross-sectional analysis in relation to sustainable food procurement, reflecting differences in overall policy support and goals. Many of the municipalities and regions reported initiatives with regard to food waste and the use of local foods, whereas initiatives with regard to reducing the climate impact of the food procurement and shift towards more plant-based menus is still in an early implementation stage. Results suggest a possible synergy effect between the use of organic food and efforts to reduce e.g., food waste and climate impact. Aspects such as the use of resources (e.g., energy and water) are not covered in this study, although these aspects are also given great importance by many of the respondents. Based on the present study, many initiatives could be integrated in municipality policies and strategies and implemented already now, e.g., serving more plant-based foods such as legumes on account of especially ruminant meat, while larger changes in meal composition would require wider discussion at the society level and documentation on e.g., nutritional adequacy and acceptability of meals among particular vulnerable citizens. Enabling factors to move sustainable food procurement forward include engaging in market dialogue, enhancing staff skills and motivation, implementing wrap-around initiatives to gain support for changes, and documenting the food composition and progress in environmental reduction based on purchase data (climate impact and other impacts such as biodiversity losses) as well as food waste quantification.

## Figures and Tables

**Table 1:** Overview of participating municipalities and regions labelled by numbers in the cross-sectional study.

Municipality (M)/Region (R)	Region Areas ^1^	Rural or Urban ^2^	Organic Objective (Y/N) ^3^	POGI Member (Y/N) ^4^	Interview Respondents
M1	1	Urban	N	N	Procurement Officer; Nutritionist; Health and care officer
M2	1	Urban	N	N	Procurement Officer; Health officer
M3	2	Urban	N	N	Procurement Officer; Health and care officer
M4	4	Urban	N	Y	Procurement Officer; Food service manager
M5	5	Urban	N	N	Procurement Controller; Food service manager
M6	2	Rural	N	N	Procurement Officer; Food service manager
M7	3	Rural	N	Y	Procurement Officer; Health and care officer; Nursing home manager
M8	3	Rural	N	N	Procurement Officer; Health and care officer; Nutritionist
M9	4	Rural	N	N	Quality officer
M10	5	Rural	N	N	Procurement Officer; Food service manager
M11	1	Urban	Y	Y	Procurement Officer; Food consultant
M12	2	Urban	Y	N	Procurement Officer; Food service manager; Health and care officer
M13	3	Rural	Y	N	Procurement Officer; Food service manager
M14	4	Urban	Y	N	Procurement Officer; Children and youth consultant
M15	5	Urban	Y	Y	Procurement Officer; Procurement Officer; Health and care officer
R1	2	-	Y	Y	Procurement officer; Food service officer
R2	4	-	(Y)	N	Procurement officer; Food service officer

^1^ A total of five Danish regions, each covering several municipalities, ^2^ Division into rural municipalities and urban municipalities [21], ^3^ Policy support and objectives for organic food procurement based on results from a survey by Organic Denmark [22], ^4^ The network organisation Partnership for Public Green Procurement (POGI) supports sustainable development by developing joint procurement goals and criteria for members to use.

**Table 2:** Key themes and subthemes identified in interviews.

Main Themes	Sub-Themes
Overall policy strategies	Overall policy support and goals
	The 17 UN SDGs as an overall framework for strategy development
	Procurement agreements and networks
Organic food	Political support for use of organic food and requirements for achievement of the Danish Organic Cuisine Label
	Organic procurement share based on institutions own initiative
	Drivers and barriers for organic food procurement
	Requirements directed at the supplier
Local and seasonal foods	Priorities for local and seasonal foods
	Drivers and barriers for local and seasonal foods
	Use of specific product groups, e.g., local vegetables in season
Climate-friendly and healthy menus	Political support towards climate reduction
	Requirements and goals towards climate reduction with regard to food procurement
	Opportunities and barriers for documentation of climate impact
	Requirements, initiatives and achievements towards more plant-based foods and less animal-based foods
	Concerns and experiences regarding consumers’ satisfaction and fulfillment of their nutritional requirements
Food waste	Requirements and goals towards reducing food waste
	Drivers, initiatives and achievements towards less food waste in the kitchens
	Requirements to reduce food waste at the supplier’s level

## Data Availability

Data available upon request.

## Supplementary Materials

The following supporting information can be downloaded at: https://www.mdpi.com/article/10.3390/foods12101975/s1, Table S1: Examples of municipalities’ requirements for sustainable food procurement, other catering practices and documentation from the two studies.

## References

[1] ICLE (2021). FOAM: Sustainable Public Procurement of Food—A Goal within Reach. Paper Written in the Framework of the EU Food Policy. https://foodpolicycoalition.eu/wp-content/uploads/2021/06/Sustainable-public-procurement-of-food-a-goal-within-reach_EU-FPC-website.pdf.

[2] Chen C., Chaudhary A., Mathys A. (2022). Dietary Change and Global Sustainable Development Goals. Front. Sustain. Food Syst..

[3] (2023). UN SDG 12 HUB: Target 12.7. Sustainable Public Procurement. https://sdg12hub.org/sdg-12-hub/see-progress-on-sdg-12-by-target/127-public-procurement.

[4] Morley A. (2021). Procuring for change: An exploration of the innovation potential of sustainable food procurement. J. Clean. Prod..

[5] Galt H., Elbrecht J. (2021). Policy Brief: Leveraging Public Procurement to Promote Sustainable Diets in the European Union.

[6] Wahlen S., Heiskanen E., Aalto K. (2012). Endorsing Sustainable Food Consumption: Prospects from Public Catering. J. Consum. Policy.

[7] Björkbom C. (2023). The EU sustainable food systems framework—Potential for climate action. NPJ Clim. Action.

[8] World Health Organization (2021). Action Framework for Developing and Implementing Public Food Procurement and Service Policies for a Healthy Diet.

[9] FAO (2021). Public Food Procurement for Sustainable Food Systems and Healthy Diets.

[10] Global Lead City Network on Sustainable Procurement (2021). Food & Catering. Global Public Procurement Factsheet.

[11] Friel S., Collin J., Daube M., Depoux A., Freudenberg N., Gilmore A.B., Johns P., Laar A., Marten R., McKee M. (2023). Commercial determinants of health: Future directions. Lancet.

[12] Kitaoka K. (2018). The National School Meal Program in Brazil: A Literature Review. Jpn. J. Nutr. Diet..

[13] European Commision (2020). A Farm to Fork Strategy—For a Fair, Healthy and Environmentally-Friendly Food System.

[14] European Public Health Alliance (2021). EPHA Contribution to the European Sustainable Food System Framework Initiative.

[15] European Commission (2021). Action Plan for the Development of EU Organic Production.

[16] (2020). Danish Ministry of Finance: Green Procurement for a Green Future—Strategy for Green Public Procurement. https://fm.dk/udgivelser/2020/oktober/groenne-indkoeb-for-en-groen-fremtid-strategi-for-groenne-offentlige-indkoeb/.

[17] Boyano A., Espinosa N., Rodriguez Quintero R., Neto B., Gama Caldas M., Wolf O. (2019). EU GPP Criteria for Food Procurement, Catering Services and Vending Machines.

[18] IPES FOOD (2019). Towards a Common Food Policy for the EU—The Policy Reform and Realignment That Is Required to Build Sustainable Food Systems in Europe.

[19] Swensson L.F.J., Hunter D., Schneider S., Tartanac F. (2021). Public food procurement as a game changer for food system transformation. Lancet Planet. Health.

[20] WHO (2023). Healthy Public Food Procurement and Service Policies. https://www.who.int/news/item/15-07-2022-the-untapped-potential-of-healthy-public-food-procurement-and-service-policies-to-support-the-repurposing-of-food-and-agricultural-policies-for-delivery-of-affordable-healthy-diets.

[21] (2022). Statistics Denmark: Municipality Groups v1:2018. https://www.dst.dk/da/Statistik/dokumentation/nomenklaturer/kommunegrupper.

[22] Organic Denmark (2021). Internal Survey: The Danish Organic Map.

[23] Doody O., Noonan M. (2013). Preparing and conducting interviews to collect data. Nurse Res..

[24] Anderson R. (1997). Thematic Content Analysis (TCA). Descriptive Presentation of Qualitative Data. https://rosemarieanderson.com/wp-content/uploads/2014/08/ThematicContentAnalysis.pdft.

[25] The City of Copenhagen (2019). The City of Copenhagen’s Food Strategy 2019.

[26] Lassen A.D., Nordman M., Christensen L.M., Trolle E. (2021). Scenario Analysis of a Municipality’s Food Purchase to Simultaneously Improve Nutritional Quality and Lower Carbon Emission for Child-Care Centers. Sustainability.

[27] Lassen A.D., Nordman M., Christensen L.M., Beck A.M., Trolle E. (2021). Guidance for Healthy and More Climate-Friendly Diets in Nursing Homes—Scenario Analysis Based on a Municipality’s Food Procurement. Nutrients.

[28] The City of Copenhagen (2021). Food Recipes.

[29] (2022). Odense Municipality: The Organization Food and Volunteering. Sustainable Food. https://www.odense.dk/mad/for-fagfolk/strategi/baeredygtig-mad.

[30] (2022). Skanderborg Municipality: Climate, Energy and Resource Policy—Green Transition in Skanderborg. https://www.skanderborg.dk/politik-og-faellesskab/groen-skanderborg/det-sker-i-kommunen/politikker-og-planer/klima-energi-og-ressourcepolitik.

[31] Ministry of Food, Agriculture and Fisheries of Denmark (2023). Organic Denmark: What Is the Organic Cuisine Label?. https://www.oekologisk-spisemaerke.dk/horeca-en.

[32] Research Institute of Organic Agriculture FiBL (2023). The World of Organic Agriculture Statistics and Emerging Trends 2023.

[33] Rose D., Heller M.C., Roberto C.A. (2019). Position of the Society for Nutrition Education and Behavior: The Importance of Including Environmental Sustainability in Dietary Guidance. J. Nutr. Educ. Behav..

[34] Basnet S., Wood A., Röös E., Jansson T., Fetzer I., Gordon L. (2023). Organic agriculture in a low-emission world: Exploring combined measures to deliver sustainable food system in Sweden. Sustain. Sci..

[35] The City of Copenhagen (2022). Annual Report 2021—Sustainable Procurement.

[36] Schleiffer M., Landert J., Moschitz H. (2022). Assessing public organic food procurement: The case of Zurich (CH). Org. Agric..

[37] WHO (2023). From Value-For-Money to Values-For-Money: Vienna’s Public Procurement Paradigm Shift. https://www.who.int/europe/news/item/06-04-2022-from-value-for-money-to-values-for-money--vienna-s-public-procurement-paradigm-shift.

[38] Daugbjerg C. (2023). Using public procurement of organic food to promote pesticide-free farming: A comparison of governance modes in Denmark and Sweden. Environ. Sci. Policy.

[39] Sørensen N.N., Sørensen M.L.K., Trolle E., Lassen A.D. (2019). Organic Food in Public Catering: How the Danish Organic Cuisine Label May Maintain Organic Food Production in the Longer Term. J. Culin. Sci. Technol..

[40] Sørensen N.N., Tetens I., Loje H., Lassen A.D. (2016). The effectiveness of the Danish Organic Action Plan 2020 to increase the level of organic public procurement in Danish public kitchens. Public Health Nutr..

[41] Lindström H., Lundberg S., Marklund P.O. (2022). Green public procurement: An empirical analysis of the uptake of organic food policy. J. Purch. Supply Manag..

[42] (2023). Sustainable Development Goals Fund: Goal 2: Zero Hunger. https://www.sdgfund.org/es/node/223.

[43] Schmitt E., Galli F., Menozzi D., Maye D., Touzard J.M., Marescotti A., Six J., Brunori G. (2017). Comparing the sustainability of local and global food products in Europe. J. Clean. Prod..

[44] Tubiello F.N., Karl K., Flammini A., Gütschow J., Obli-Laryea G., Conchedda G., Pan X., Qi S.Y., Heiðarsdóttir H.H., Wanner N. (2022). Pre- and post-production processes increasingly dominate greenhouse gas emissions from agri-food systems. Earth Syst. Sci. Data.

[45] Galloway C., Devine S., Parison J., Jones H.A. (2023). Procurement from local producers for food service in primary and secondary school settings: A scoping review. Health Promot. J. Aust..

[46] Lassen A.D., Christensen L.M., Trolle E. (2020). Development of a Danish Adapted Healthy Plant-Based Diet Based on the EAT-Lancet Reference Diet. Nutrients.

[47] Trolle E., Nordman M., Lassen A.D., Colley T.A., Mogensen L. (2022). Carbon Footprint Reduction by Transitioning to a Diet Consistent with the Danish Climate-Friendly Dietary Guidelines: A Comparison of Different Carbon Footprint Databases. Foods.

[48] Speck M., Wagner L., Buchborn F., Steinmeier F., Friedrich S., Langen N. (2022). How public catering accelerates sustainability: A German case study. Sustain. Sci..

[49] Danish Veterinary and Food Administration (2021). The Official Dietary Guidelines—Good for Health and Climate.

[50] Danish Veterinary and Food Administration (2021). Advice on Food and Drink When You Are over 65 Years Old.

[51] Pedersen A.N., Ovesen L. (2015). Recommendations of the Danish Institution Diet.

[52] Danish Veterinary and Food Administration (2021). Dietary Guidelines for Meals in Kindergartens, Schools and Workplaces.

[53] Pintado T., Gado-Pando G. (2020). Towards more sustainable meat products: Extenders as a way of reducing meat content. Foods.

[54] Boukid F., Zannini E., Carini E., Vittadini E. (2019). Pulses for bread fortification: A necessity or a choice?. Trends Food Sci. Technol..

[55] The Aarhus City Council (2022). Minutes from the City Council Meeting on 27 April 2022. https://dagsordener.aarhus.dk/vis?id=696b9739-f53a-40fd-bf70-1ff5897d387b.

[56] Lassen A.D., Christensen L.M., Spooner M.P., Trolle E. (2019). Characteristics of Canteens at Elementary Schools, Upper Secondary Schools and Workplaces that Comply with Food Service Guidelines and Have a Greater Focus on Food Waste. Int. J. Environ. Res. Public Health.

[57] Sundin N., Malefors C., Danielsson M., Hardiyanti M., Persson Osowski C., Eriksson M. (2023). Investigating goal conflicts in menu planning in Swedish school catering on the pathway to sustainable development. Resour. Conserv. Recycl..

[58] Malefors C. (2022). Doctoral Thesis: Food Waste Reduction in the Public Catering Sector.

[59] WHO Regional Office for Europe (2022). How together We Can Make the World’s Most Healthy and Sustainable Public Food Procurement.

[60] (2023). Procura+ Network: Procura+ Awards 2021: Copenhagen & Odense Denmark—Beyond Organic Food Procurement According to the Sustainable Development Goals. https://procuraplus.org/dev/awards/awards-2021/.

[61] WHO Regional Office for Europe (2021). Healthy and Sustainable Diets. Report of an Expert Meeting on Healthy and Sustainable Diets. A Workshop to Share Challenges, Identify Knowledge Gaps and Receive Feedback, 24–25 March 2021.

[62] FAO (2018). Title of Practice. Copenhagen: Organic Conversion in Public Kitchens.

[63] Lassen A., Thorsen A.V., Trolle E., Elsig M., Ovesen L. (2004). Successful strategies to increase the consumption of fruits and vegetables: Results from the Danish ‘6 a day’ Work-site Canteen Model Study. Public Health Nutr..

[64] Rosewarne E., Chislett W.K., McKenzie B., Mhurchu C.N., Boelsen-Robinson T., Blake M., Webster J. (2022). Understanding Enablers and Barriers to the Implementation of Nutrition Standards in Publicly Funded Institutions in Victoria. Nutrients.

[65] European Public Health Alliance (2022). Manifesto for Establishing Minimum Standards for Public Canteens across the EU.

