# Supplementary material for: Current Practices and Opportunities for More Sustainable Public Food Procurement: A Qualitative Study among Danish Municipalities and Regions
Source: Foods. 2023 May 12;12(10):1975. doi: 10.3390/foods12101975 (PMC10217290; doi:10.3390/foods12101975)
Supplement: Supplementary file 1 [file foods-12-01975-s001.zip › foods-2366414-supplementary.pdf]

foods

Supplementary Materials

Current Practices and Opportunities for More Sustainable Public Food Procurement

Anne Dahl Lassen, Anne Vibeke Thorsen and Ellen Trolle

Table S1. Examples of municipalities' requirements for sustainable food procurement, other catering practices and documentation from the two studies

	Examples of sustainable procurement criteria	Examples of criteria for sustainable catering practice	Examples of documentation
Organic food	Requirements for a fully organic variety<br>Requirements for specific product groups of organic quality<br>Setting up shopping records with just organic products	Minimum requirements for proportion of organic procurement, e.g., min 60 %<br>Use of specific product groups of organic quality	Achievement of the Danish Organic Cuisine Label available in three versions according to organic share; Gold (90-100%), Silver (60-90%) and Bronze (30-60%)<br>Documentation of organic procurement for a certain period provided from the supplier
Local and seasonal food	Requirements for certain varieties of seasonal food<br>Possibility of separate (smaller) food procurement<br>Requirements for products with short supply chain and seasonal varieties to ensure variation and product diversity	Targets for a minimum share of purchased food that must be from local sources<br>Use of specific product groups, e.g. local seasonal vegetables as well as demand for different varieties	The supplier provides a list of manufacturers of specific products<br>Documentation of consumption during a certain period based on food procurement
Climate-friendly and healthy menus	Information on climate impact at food level from suppliers (climate labelling in the long run)<br>Requirements regarding supply of plant products such as legumes and nuts<br>Competitive prices for more climate-friendly foods that the public kitchens should serve more of, such as legumes and nuts<br>Use of behavioral design when ordering to promote climate-friendly and healthy foods	Follow healthy and climate-friendly dietary guidelines<br>Requirements regarding the degree of plant-based meal composition, e.g., smaller portion sizes of meat (meat-free days, etc.) including less beef (e.g., less than ¼ of the total meat consumption should be beef) as well as more legumes, nuts, seeds, etc.<br>Requirements regarding nudging towards more plant-based meals	Information on total climate impact and allocated on different food groups (total and at unit level)<br>Documentation regarding compliance with recommendations or nutrition calculations of recipes and meals<br>Adaption of existing quality standards for, e.g., food delivery (meal on wheels) (amount of meat, fish, legumes, etc.)
Minimizing food waste	Requirements for the suppliers' handling of waste, e.g., the supplier must have policies for minimizing food waste<br>Possibility of highlighting foods when purchasing in order to avoid food waste	Requirements for reducing food waste, e.g., 30 %<br>Requirements regarding use and recycling of raw materials to reduce food waste through planning, utilization of the entire raw material, recycling of leftovers, portion size and dialog with the customers etc.	Obtain an account of the suppliers' food waste<br>Food waste quantification and plans for handling

Other sustainable criteria at product level	Requirements on fair and ethical products, e.g., Fairtrade branded cocoa, coffee beans and bananas<br>Requirements for animal welfare, sustainable/certified fish etc.<br>Requirements for phasing out non-certified palm oil and soybean<br>Criteria for processed products – e.g., keyhole label, salt content, etc., ingredients, etc.	Minimum share of certified products within the product group, e.g., min. 50 % Fairtrade coffee, tea, cocoa<br>Restriction of highly processed food	Documentation of consumption for a given period based on food procurement
